# Aging of the Immune System: Research Challenges to Enhance the Health Span of Older Adults

**DOI:** 10.3389/fragi.2020.602108

**Published:** 2020-10-15

**Authors:** Laura Haynes

**Affiliations:** UConn Center on Aging, University of Connecticut School of Medicine, Farmington, CT, United States

**Keywords:** inflammaging, cellular senescence, health span, geroscience hypothesis, aging immune system

The world’s population is aging, which brings about huge scientific challenges. Since the biological process of aging is by far the greatest risk factor for most chronic diseases, understanding the molecular and cellular mechanisms by which aging leads to these conditions is of vital importance to increase the health span of older adults.

One of the most prominent biological systems to face the impacts of aging is the immune system, with age-related changes in immunity being responsible for an increase in susceptibility to infectious diseases and a decrease in the effectiveness of vaccinations. In addition, the innate immune system is responsible for one of the main characteristics of aging, namely, the increase in low-grade inflammation known as inflammaging. Five research challenges present themselves: the origins and impact of age-related inflammation, the impact of senescence on immunity, age-related changes in immune function, the effects of decreased immune function on infection and vaccination, and strategies to overcome declining immunity. These are all especially important at this time, since aging is a major risk factor for the development of severe complications from infectious diseases such as COVID-19, influenza, and bacterial pneumonia.

## Aging and inflammation (Inflammaging)

One of the main characteristics of aging is chronic activation of the immune system that results in low-grade inflammation which is mainly driven by macrophages (Franceschi et al., 2000). The age-related mechanisms responsible for driving this inflammation include cellular senescence, mitochondrial dysfunction, defective autophagy, activation of the inflammasome, activation of the DNA damage response, changes in the composition of the microbiome, and chronic infections such as those caused by human cytomegalovirus (CMV) (Vitale et al., 2013; Pawelec, 2014; Heath and Grant, 2020). The mediators of this systemic inflammation are pro-inflammatory cytokines such as interleukin-6 and tumor necrosis factor alpha, which increase with increasing age (Bruunsgaard, 2006; Maggio et al., 2006). This chronic inflammation is a highly significant risk factor for both morbidity and mortality in older adults (Franceschi et al., 2000) and is hypothesized to be a driver of age-related diseases such as type 2 diabetes, Alzheimer’s disease, atherosclerosis, osteoarthritis, cancer, and hypertension (Freund et al., 2010; Childs et al., 2017; He and Sharpless, 2017).

While researchers have gained a better understanding of inflammaging, other impacts of inflammaging remain to be explored, including its influence on adaptive immune responses such as vaccine-induced protective immunity, T-cell differentiation, and susceptibility to complications following respiratory infections such as COVID-19 and influenza.

## Senescence

Cellular senescence is characterized by a state of essentially irreversible proliferative arrest caused by potentially oncogenic stressors and is now recognized as an important tumor suppression mechanism (Braig et al., 2005; Michaloglou et al., 2005; Collado and Serrano, 2010). Importantly, senescent cells secrete a myriad of factors which have been called the “senescence-associated secretory phenotype” (SASP). The main goal of SASP production is to activate innate immune cells, such as macrophages, to clear the SASP-producing senescent cells by phagocytosis and, thus, prevent tumor development. The SASP includes growth factors, pro-inflammatory cytokines, chemokines, and proteases, all of which can contribute to inflammaging (Coppe et al., 2008; Freund et al., 2010). With increasing age, senescent cells are not cleared as efficiently by the innate immune system, causing them to accumulate and increasing SASP levels (Coppe et al., 2008; Zhu et al., 2014; Xu et al., 2018). Furthermore, senescent cell accumulation in fat also increases with aging, linking senescence to age-related changes in metabolism (Xu et al., 2015). Senescent cells and SASP have dramatic impacts on disease states, including loss of physical function, osteoarthritis, frailty, and loss of insulin sensitivity (LeBrasseur et al., 2015; Xu et al., 2015; Xu et al., 2018). Recent studies have shown that clearance of these senescent cells in transgenic mouse models or by senolytic drug treatment can alleviate senescence-associated disease states (Xu et al., 2015; Xu et al., 2018). In addition, a 2019 study has also shown that deletion of senescent cells enhances T-cell proliferation (Palacio et al., 2019).

Taken together, these studies are encouraging and support the geroscience hypothesis which states that most, if not all, age-related chronic diseases can be alleviated by interventions that retard the aging process and/or senescence. Importantly, the precise role that senescence and SASP play in age-related changes in innate and adaptive immunity remains relatively unexplored and is a potentially important area of investigation.

## Age-Related Changes in The Immune Response

While the impact of aging on both innate and adaptive immunity has been well documented, the mechanistic causes are not well understood. The age-related changes in immune function are most likely due to a combination of intrinsic cell aging and the impact of the senescent/aging environment on proliferation and differentiation in response to antigenic stimulation. In addition to age-related changes in cells of the immune system, there are changes in chemokine localization and the microarchitecture of both lymph nodes and spleen that can impact cell trafficking and encounters with cognate antigen (Thompson et al., 2017; Turner and Mabbott, 2017; Masters et al., 2018; Masters et al., 2019). Innate immune cells contribute to inflammaging by producing cytokines that are associated with chronic inflammation, but other important functions of innate immune cells such as phagocytosis, antigen uptake and presentation, migration, and bactericidal activity are diminished with aging (Henry et al., 2011). With regard to adaptive immunity, CD4 and CD8 T cells and B cells all show age-related changes in function. Due to early life thymic involution and a lifetime of antigenic exposure, memory phenotype T cells predominate in older individuals (Cossarizza et al., 1996; Linton and Dorshkind, 2004). Additionally, chronic infections such as CMV infections contribute to the accumulation of highly differentiated memory CD8 T cells that exhibit characteristics of replicative senescence (Ouyang et al., 2003; Pawelec and Gouttefangeas, 2006). The age-related accumulation of these terminally differentiated CD8 T cells serves to constrict the immune repertoire and has also been associated with the impaired immune response to vaccinations and novel infections, such as SARS-CoV-2, observed in older adults (Akbar and Fletcher, 2005; McElhaney et al., 2012). With regard to CD4 T cells, age-related changes in function include diminished proliferative capacity, inappropriate T helper subset differentiation, and an increase in the percentage of regulatory T cells (Moro-Garcia et al., 2013; Lorenzo et al., 2018). B cell function is also significantly impacted by aging: several B-cell biomarkers of aging have been characterized and are related to reduced class-switch recombination and somatic hypermutation of immunoglobulin genes, which ultimately result in reduced antibody production and function following vaccination or infection (Blomberg and Frasca, 2013).

The mechanisms responsible for these age-related changes in both innate and adaptive immunity remain to be explored, and a more thorough understanding could help us devise better strategies to overcome them.

## How Age-Related Changes Impact Infection and Vaccination

Because of the abovementioned age-related changes in innate and adaptive immune function, older adults exhibit increased susceptibility to infections such as influenza, COVID-19, and bacterial pneumonia. Influenza is responsible for up to 500,000 deaths per year worldwide, with two-thirds of these occurring in adults over 65 years of age (Paget et al., 2019). Older adults are also more susceptible to COVID-19 and its complications (Nanda et al., 2020). In the U.S., 80% of COVID-19 deaths occur in people >65 years of age, and mortality rates for those >80 years of age can be as high as 50% (Powell et al., 2020). The incidence of bacterial pneumonia in adults in the U.S. >65 years of age is more than 4 times higher than that found in adults aged <45 years, and hospitalization rates for elderly patients is highest for the oldest groups (Henig and Kaye, 2017).

Not only are older adults more susceptible to these infections, but they are also more susceptible to complications during these infections, in part because of the many co-morbidities associated with increasing age (Keilich et al., 2019). Vaccinations are strongly recommended for older adults to protect them from both influenza and bacterial pneumonia, but because of age-related changes in immune function, vaccine efficacy and effectiveness decline with age (Ciabattini et al., 2018). Further understanding of how aging of the immune system results in increased susceptibility to infectious diseases and decreased vaccine efficacy will contribute greatly to improving the health span of older adults.

## Strategies to Overcome Age-Related Changes in Immunity

Research continues to discover approaches to overcoming age-related declines in immunity. Novel approaches to specifically target the immune system as well as geroscience-directed approaches, which target the underlying causes of aging, should provide us with exciting new therapies to extend the health span of older adults.

Several approaches are already in use or are undergoing testing. First, vaccines specifically formulated for older adults are now available and have been shown to provide greater protection from viral infections such as influenza. These vaccines have higher concentrations of antigen or are formulated with adjuvants to boost aging immune responses (McElhaney et al., 2020). Second, approaches that alter metabolism such as treatment with metformin (Barzilai et al., 2016) or mTOR inhibitors (Mannick et al., 2018) could help improve immunity and resistance to infectious diseases in older adults. Last, senolytics, which are a novel class of drugs that target the destruction of senescent cells, have been shown to alleviate age-related diseases in animal models and could possibly also improve aged immune function (Xu et al., 2018).

Continued research in this field will help discover other novel approaches which will help enhance and extend the life span of older adults.

## Conclusion

Responding to the unanswered questions in the research challenges outlined, the aim of the specialty section *Aging and the Immune System* (part of *Frontiers in Aging*) is to contribute to the overall understanding of aging and immunity with a focus on the translational aspects that may improve human health span.

## Author Contributions

The author confirms being the sole contributor of this work and has approved it for publication.

## Conflict of Interest

The author declares that the research was conducted in the absence of any commercial or financial relationships that could be construed as a potential conflict of interest.
